# Comprehensive screening of genes resistant to an anticancer drug in esophageal squamous cell carcinoma

**DOI:** 10.3892/ijo.2015.3085

**Published:** 2015-07-16

**Authors:** MAI TSUTSUI, HIROFUMI KAWAKUBO, TESTSU HAYASHIDA, KAZUMASA FUKUDA, RIEKO NAKAMURA, TSUNEHIRO TAKAHASHI, NORIHITO WADA, YOSHIRO SAIKAWA, TAI OMORI, HIROYA TAKEUCHI, YUKO KITAGAWA

**Affiliations:** Department of Surgery, School of Medicine, Keio University, Shinjuku-ku, Tokyo 160-8582, Japan

**Keywords:** esophageal cancer, drug resistance, transposon, gene, cisplatin

## Abstract

Drug resistance to chemotherapy is a major issue in esophageal cancer management. Drug resistance may be mediated by genetic changes in the tumor; therefore, the identification of gene mutations may lead to better therapeutic outcomes. We used a novel method involving transposons to screen and identify drug-resistant genes. Transposons are DNA sequences that move from one location on the gene to another. A modified piggyBac transposon was designed as an insertion mutagen, and a cytomegalovirus (CMV) promoter sequence was added to induce strong transcription. When the transposon is inserted to the upstream of a certain gene, the gene will be overexpressed while when intserted down or intragenically, it will be downregulated. After establishing a transposon-tagged cell library, we treated cell lines derived from esophageal squamous cell carcinomas (ESCC) [Tohoku esophagus (TE)] with cisplatin (CDDP). We performed splinkerette PCR and TOPO cloning on the resistant colonies. Bacterial colonies were sequenced, and next-generation sequencing was used to identify the overexpressed/downregulated sequences as candidate genes for CDDP resistance. We established 4 cell lines of transposon-tagged cells, TE4, 5, 9 and 15. We treated the two relatively viable cell lines, TE4 and TE15, with CDDP. We identified 37 candidate genes from 8 resistant colonies. Eight genes were overexpressed whilst 29 were downregulated. Among these genes was Janus kinase 2 (*JAK2*) that is implicated in the progression of myeloproliferative neoplasms. We identified 37 candidate genes responsible for CDDP resistance in the two cell lines derived from ESCC cells. The method is inexpensive, relatively simple, and capable of introducing activating and de-activating mutations in the genome, allowing for drug-resistant genes to be identified.

## Introduction

Esophageal cancer is responsible for more than 410,000 deaths per year around the world and is the seventh leading cause of death according to the WHO 2008 database. There are two different types of esophageal cancers, adenocarcinoma and squamous cell carcinoma.

Adenocarcinoma is the most common type of esophageal cancer in Western societies whilst squamous cell carcinoma is more common in Eastern societies, with both being life-threatening diseases unless diagnosed at an early stage. Despite the pathological difference, the clinical presentation and management options are broadly similar (1). Multimodal therapies are necessary, and for advanced cancer, chemotherapy is one of the major treatment options in both pathological types along with radiotherapy and surgery. At present, the combination of cisplatin (CDDP) and 5-fluorouracil (5-FU) is widely used as a standard regimen. The results of the Radiation Therapy Oncology Group (RTOG) study (2) showed that the standard radiation dose for patients treated with concurrent 5-FU and CDDP chemotherapy is 50.4 Gy which has now been widely adopted in Western countries. Based on the results of a randomized study in Japan (Japan Clinical Oncology Group: JCOG 9204 and JCOG 9907), the standard therapy in Japan for stage II/III thoracic squamous cell carcinoma is preoperative chemotherapy with CDDP and 5-FU followed by esophagectomy (3,4).

While there are patients that respond well to the drugs, some patients suffer from drug resistance in the course of treatment (5–7). When drug resistance emerges, chemotherapy can cause more harm than benefit to the patients, leading to a poor quality of life (QOL) and mortality. In particular, CDDP is classified as a highly emetic drug causing severe side effects (8). Therefore, to be able to predict drug resistance is useful. There have been some studies indicating that drug resistance may be mediated by genetic changes. In the case of esophageal squamous cell carcinoma (ESCC) and CDDP, the overexpression of multidrug-resistant protein gene (MRP1/2) (9,10) and the zinc ribbon domain-containing-1 (ZNRD1) (7) have been reported to be involved in this resistance. A number of methods have been used to identify genes involved in resistance to chemotherapy, including microarray, expression profiling, cDNA library screens, RNAi library screens and small molecule inhibitor screens (11–13).

Here, we performed a novel procedure using transposons as a comprehensive method for screening resistant genes developed by Li *et al* (14). The approach uses a modified piggyBac (PB) (15) and Sleeping Beauty (SB) transposon to generate libraries of mutagenized cells, each containing transposon insertions that randomly activate nearby gene expression. The PB transposon randomly inserts into the host genome aided by the enzyme transposase, requiring only TTAA as its integration sites. Li *et al* found the multidrug-resistant gene *MDR1/ABCB1* as a resistant gene for paclitaxel by this method from three different cell lines. We adopted this method because it is possible to generate stable, resistant clones relevant to specific cell lines by this method. Our aim in the present study was to further seek CDDP-resistant genes in ESCC cells, with the expectation of better patient QOL and survival and further therapy. Application of this method by overexpressing and downregulating target genes may also prove to be effective in gene therapy.

## Materials and methods

**Figure 1:**
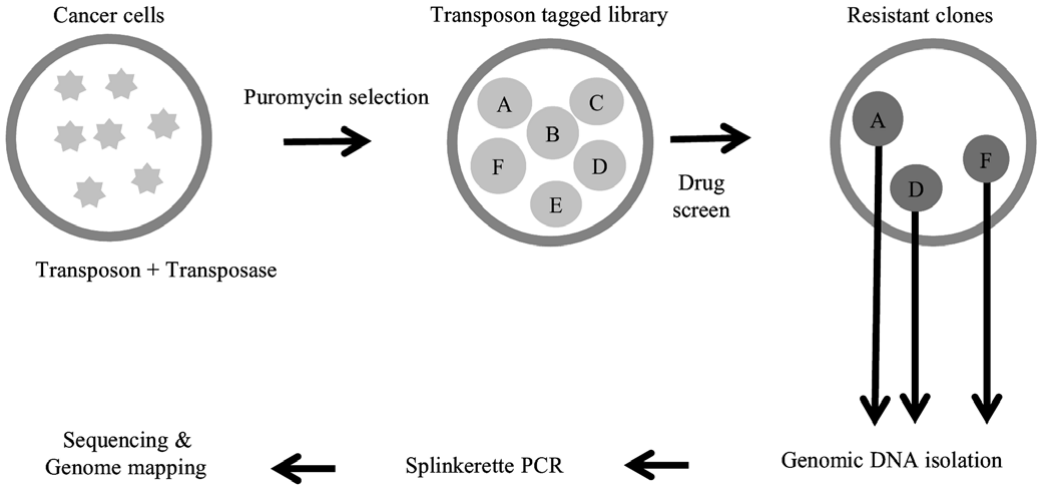
Overview of the experimental system used for screening drug-resistant genes. Cancer cells were first transfected and then selected with puromycin to confirm the transfection. CDDP was added for drug screening, and the resistant colonies were harvested to isolate genomic DNA. The insertion sites were identified by sprinkerette PCR and resistant genes were detected.

The overview of the experimental system is shown in Fig. 1.

### Plasmid construction

**Figure 2:**
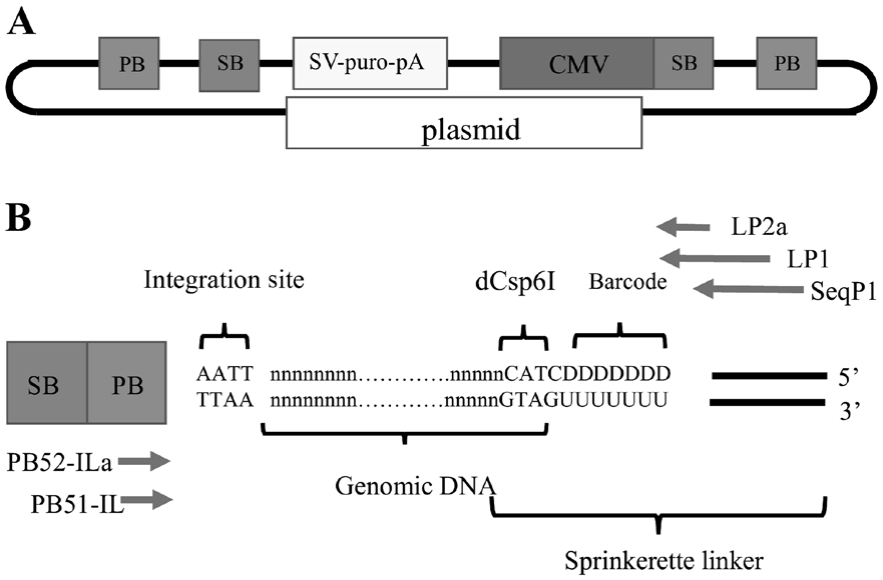
(A) Diagram of PB plasmid pPB-SB-CMV-puro-SD. The cytomegalovirus (CMV) enhancer and promoter sequence was added. (B) Splinkerette PCR template for insersion site detection.

Transposon plasmid PB-SB-PGK-neo-bpA and transposase plasmid pCMV-PBase were kindly provided by Dr Li Chen from Massachusetts General Hospital, Harvard Medical School, Boston, MA, USA. This plasmid was designed as an insertion mutagen that disrupts the structure of the inserted host gene. Several changes were made in PB-SB-PGK-neo-bpA to convert it to an activation mutagen. The plasmid was first digested with *Hin*dIII restriction enzyme and calf intestinal phosphatase, and ligated with a PAR-amplified fragment containing the CMV enhancer and promoter sequence to activate genes located adjacent to transposon insertion sites. The splice donor from rabbit β-globlin intron was next used to make pPB-SB-CMV-neo-SD. The pPB-SB-CMV-neo-SD plasmid was then digested with *BgI*II and *Xma*l to remove the PGK-Neo-bpA cassette and was ligated with a PCR-amplified SV40-driven puromycin cassette to provide a rapid selection marker to identify successful integrants. The final plasmid was sequence-verified and named pPB-SB-CMV-puro-SD (Fig. 2A). By this method, it occurs that transposon insertion upstream of a certain gene sequence leads to upregulation of the gene while intragenic insertions downstream will lead to downregulation.

### Cells

Human ESCC cell lines [Tohoku esophagus (TE) 4, 5, 9 and 15 (16,17)] were obtained from the Cell Resource Center for Biomedical Research Institute of Development, Aging and Cancer, Tohoku University (Sendai, Japan). TE4 and 15 cells were derived from well-differentiated squamous cell carcinomas while TE5 and 9 cells were derived from undifferentiated cells. The cell lines were cultured in Dulbecco's modified Eagle's medium (DMEM) supplemented with 10% fetal bovine serum (FBS; Invitrogen, Carlsbad, CA, USA) and penicillin-streptomycin solution (penicillin 10,000 U/ml, streptomycin 10,000 μg/ml; Nacalai Tesque, Inc., Kyoto, Japan). In all experiments, cells were cultured at 37°C in a humidified atmosphere consisting of 5% CO_2_/95% air. We verified that TE4, 5, 9 and 15 cells possess cisplatin sensitivity.

### Small scale cell line transfection and efficiency

First, we confirmed the establishment of transposon-tagged cells on a small scale. In order to determine the concentration of puromycin required for selection, we seeded 1×10^3^ cells in a 96-well plate, added puromycin from day 2, cultured the cells for 6 days and then used the Cell Count^®^ reagent to measure cell viability. The absorbance of the solution was read at 450 nm using a microtiter plate reader (Becton-Dickinson, Franklin Lakes, NJ, USA). Experiments were performed 16-fold. After pulsed exposure, the IC_50_ was calculated as the percentage of control cultures which were not exposed to cisplatin using an interpolated logarithmic concentration curve.

After determining the concentration of puromycin required for selection, we placed 1×10^5^ cells to each of two 10-cm dishes in regular medium. On day 2, we set up 2 aliquots of 28.8 μl FuGENE 6 (Roche) and 0.6 ml SF-medium (OPTI-MEM). In aliquot 1 (the positive control), we added 4.8 μl of transposon plasmid pPB-SB-CMV-puro-SD1 and 4.8 μl transposase plasmid pCMV-PBase. In aliquot 2 (the negative control), we added 4.8 μl of transposon plasmid pPB-SB-CMV-puro-SD1 and 6.14 μg pcDNA 3.2. On day 3, we trypsinized the cells and plated a dilution series of 1, 1/2, 1/4, 1/8, 1/16, and 1/32×10^5^ cells in 6-well plates in the presence of puromycin. We cultured the cells until colonies were visible and stained the colonies with Diff-Quik stain^®^ to determine the efficiency of transfection.

### Cell line transfection for library construction

To make a large scale library, 1×10^7^ cells were plated overnight in four T175 flasks at a cell density of 1×10^5^ cells per ml. On day 2, after the medium was changed, cells were co-transfected with 48 μl pPB-SB-CMV-puro-SD and 48 μl pCMV-PBace plasmids using 288 μl FuGENE 6 and 6 ml SF-medium. On day 4, the medium was replaced, and cells were treated with fresh media with puromycin and cultured for an additional 12–15 days. Cells surviving the antibiotic treatment were harvested and preserved as transposon-tagged prescreened libraries. Two million tagged cells per tube were stored in a −80°C refrigerator with the cell banker agent for future experiments.

### Cisplatin screen

CDDP was purchased from Yakult Honsha Co., Ltd. (Tokyo, Japan) and stored at a concentration of 0.5 mg/ml at room temperature. A total of 2.5×10^5^ transposon-tagged cells from each library were plated in 100-mm tissue-culture plates for drug treatment. Native, untagged cells were similarly plated as a study control. Dosages were chosen as to sufficiently kill all parental cells within 1 week. Cells were treated until CDDP-resistant colonies were visible. Treatment time varied among cell lines depending on proliferation rates and usually took between 10 days and 4 weeks. Cells were then harvested as resistant clones. To isolate resistant clones, colonies were picked from the drug-treated plates using 1000-μl blue tips into 96-well plates, followed by 24-well plates, and then they were expanded in 6-well plates in the presence of puromycin and CDDP. Cell clones exhibited stable resistance to both CDDP and puromycin and continued to grow when retreated after 2 weeks of culture in both agents. To harvest resistant pools, cells from the CDDP-treated plates were trypsinized and re-plated in the presence of puromycin and CDDP for at least one more week to remove any remaining non-resistant cells.

### Splinkerette PCR and nextgen sequencing for insertion site detection

Genomic DNA was harvested from samples using a DNeasy Blood & Tissue kit (Qiagen). Insertion sites can be detected by splinkerette PCR, a modified version of ligation-mediated PCR. We digested 170 ng of genomic DNA with 2 U of Csp6I (Fermentas) at 37°C for 2 h, and ligated with 1 μl T4 DNA ligase for 5 min at room temperature followed by 10 min at 70°C. The ligated sample was amplified with primers LP1 and PB51-IL in a 100 μl PCR reaction. Primer LP1 matches the linker sequences, whilst primer PB51-IL matches the transposon sequences. The following thermo-cycle conditions were used: 3 min/94°C, 10 cycles of 15 sec/94°C; 30 sec/72°C with −1°C touchdown/cycle; 1 min/72°C, 20 cycles of 15 sec/94°C; 30 sec/62°C; 1 min/72°C and 20 min/72°C. One microliter of the first PCR product was re-amplified in a 50-μl PCR using nested primers LP2a and PB52-ILa that contain Illumina single-end reaction adapter sequences for binding to the flowcell. Conditions for thermo-cycling were similar to that of the first PCR with 10 touchdown cycles and 10 regular cycles. Amplified products were purified using QIAquick PCR Purification kit (Qiagen) (Fig. 2B). We checked the concentration and purity of the DNA using a Nano Drop^®^ after the extraction of genomic DNA, 2nd PCR, and plasmid DNA purification to exclude inadequate samples.

### TOPO cloning and sanger sequencing

Nested PCR products of resistant clones were prepared as above and cloned into vector pCR2.1-TOPO (Invitrogen). Bacterial colonies were then sequenced using primer PB5-ILseq from the transposon side. Insertion sites were aligned using the BLAST function of the National Library of Medicine (http://blast.ncbi.nlm.nih.gov/Blast.cgi). We included a maximum of 15 genes that had either highly similar sequences (megablast) or somewhat similar sequences (blastn) as candidate genes for each colony.

### Real-time quantitative PCR analysis

Cells were harvested, and total RNA was extracted with an RNeasy kit (Qiagen, Valencia, CA, USA). The concentration of total RNA was determined using a NanoDrop ND-1000 (NanoDrop Technologies, San Diego, CA, USA). cDNA was synthesized from total RNA using a High Capacity RNA to cDNA kit (Life Technologies, MD, USA) according to the manufacturer's protocol. PCR reaction mixes were prepared using the template of cDNA samples and Fast CYBR Green Master Mix (Life Technologies), whilst the expressions of human *JAK2* and *GAPDH* were analyzed using ViiA7 Real-time PCR system (Life Technologies). The PCR-amplification primers for *JAK2* were as follows: forward primer, 5′-GAGCCTATCGGCATGGAATA-3′; and reverse primer, 5′-ACTGCCATCCCAAGACATTC-3′, generating a 160-bp amplicon. The housekeeping gene *GAPDH* was quantified with the following primers: 5′-CGAGATCCCTCCAAAATCAA-3′ and antisense 5′-TGTGGTCATGAGTCCTTCCA-3′. The thermal cycling reaction included incubation at 95°C for 20 sec, 40 cycles of 95°C for 3 sec and 60°C for 30 sec. Data were collected using analytical software (Applied Biosystems). The expression level of each gene was determined using the ΔΔCT method.

## Results

### Determination of transposon efficiency

**Figure 3:**
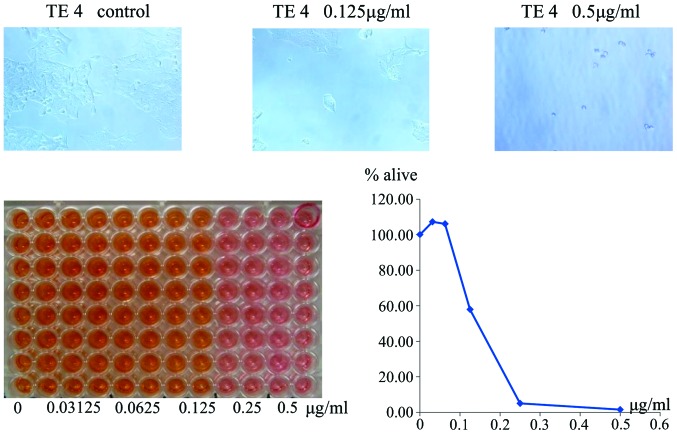
Puromycin concentration. The concentration of puromycin used for selection was verified with wild-type TE4 cells. The concentration was the same for TE15 cells.

**Figure 4:**
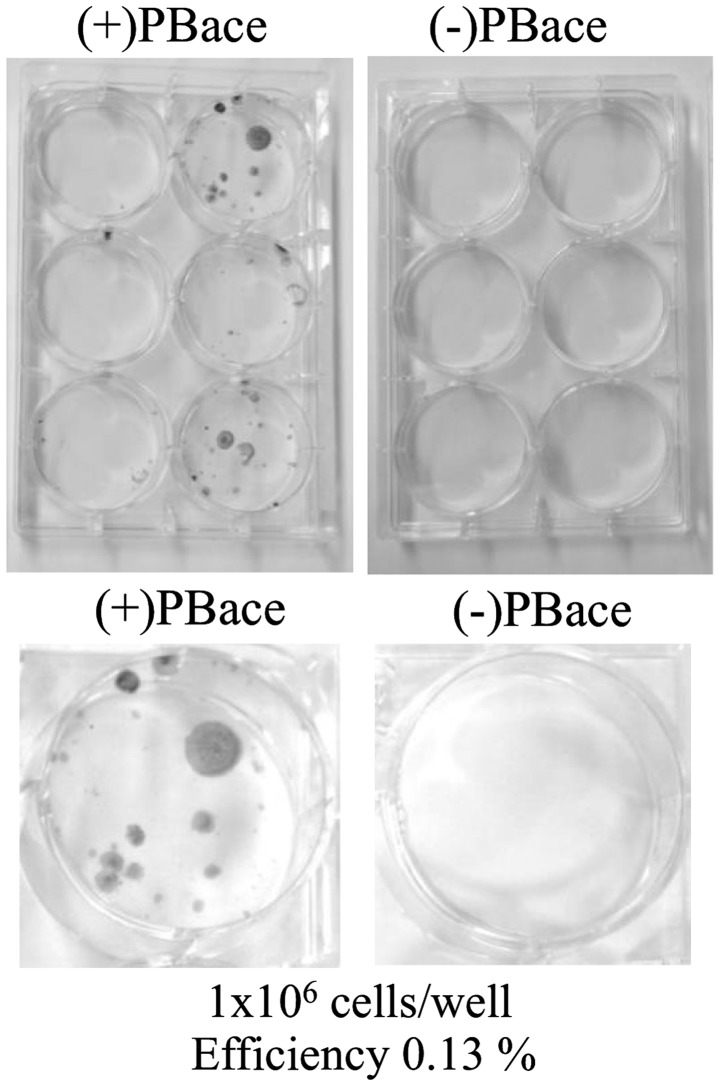
Efficiency of transfection in TE4 cells. Cells were transfected with the PB plasmid in the presence (+PBase) or absence (-PBase) of a transposase plasmid followed by puromycin treatment. The efficiency of transfection in TE4 cells was 0.13%.

In order to confirm the establishment of transposon-tagged cells, we first started from a small scale library construction. We used the TE cells to select the required concentration of puromycin. We seeded 1×10^3^ cells each in a 96-well plate and added puromycin from day 2, cultured cells for 6 days, and then used the Cell Count reagent to investigate the cell viability. The puromycin concentration for screening used was 0.5 μg/ml in TE4 cells (Fig. 3). Next cells were co-transfected with pPB1-SB-CMV-puro-SD1 and a plasmid expressing piggyBac transposase and selected for puromycin resistance. After being co-transfected with transposase, transposons were integrated into cells at a frequency of 0.13% from the original starting population of cells (Fig. 4). We repeated this efficacy experiment for 6 additional plates, resulting in similar results, with an efficiency ranging from 0.04 to 0.13%.

### Large scale acquirement of tagged cells

After the small scale confirmation, we established a large scale cell library in 4 cell lines, including TE4, 5, 9, and 15. We were able to establish tagged cells with the same puromycin concentration (0.5 μg/ml).

### Cisplatin resistance screen

**Figure 5:**
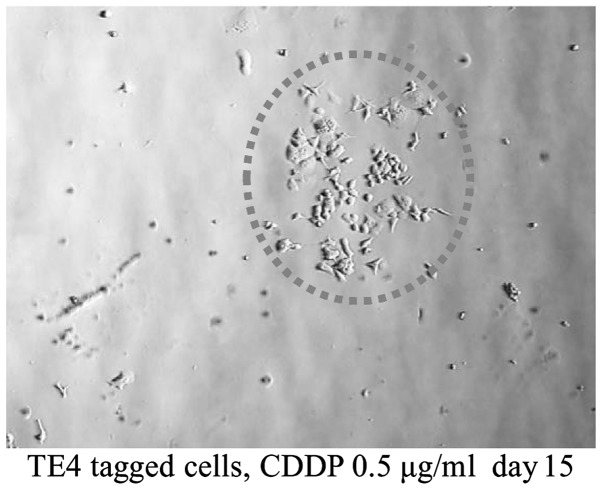
CDDP-resistant colonies. CDDP-resistant colonies were observed 10–12 days post drug addition. The CDDP concentration for TE4 cells was 0.5 μg/ml and 0.25 μg/ml for TE15 colonies.

TE4- and 15-tagged cells were chosen for CDDP drug screening because they had a higher proliferation rate compared to the TE5- and 9-tagged cells. Both wild-type and tagged cells were treated with CDDP at specified concentrations. We started from 1,000 cells per well in 96-well plates with a concentration ranging from 0 to 100 μg/ml, narrowed the range from 0 to 3 μg/ml in 2.5×10^5^ cells, and acquired CDDP-resistant colonies of concentration of 0.5 μg/ml for TE4 (Fig. 5) and 0.25 μg/ml for TE15 tagged cells. In total, we found 20 CDDP-resistant colonies from transposon-tagged TE4 cells and 31 colonies from TE15 cells. As for the wild-type TE4 and TE15 cells, no colonies were observed under the CDDP exposure.

### Identification of transposon insertion sites and candidate genes

Insertion sites from isolated single colonies were identified by sprikerette PCR, cloning of DNA flanking transposons into TOPO vectors, and Sanger sequencing. We performed agarose gel electrophoresis to confirm that DNA sequences were amplified. From the 51 colonies obtained, 39 colonies were sequenced, resulting in 8 colonies with 37 candidate genes for CDDP resistance. Eight of these genes were overexpressed whilst the remaining 29 were downregulated as shown in Table I. We performed the last step of sequencing at least twice to ensure accuracy.

### JAK2 downregulation for CDDP resistance

Among the 37 candidate genes, we focused on the downregulation of Janus kinase 2 (*JAK2*) which was identified from one of the resistant colonies of the TE4-tagged cells (cell line TE4-2). We initiated the frozen TE4-2 tagged cells and cultured them in 6-well plates to confluence in the presence of DMEM + 0.5 μg/ml CDDP and puromycin. We also cultured wild-type TE4 cells in the presence of DMEM for comparison.

**Figure 6:**
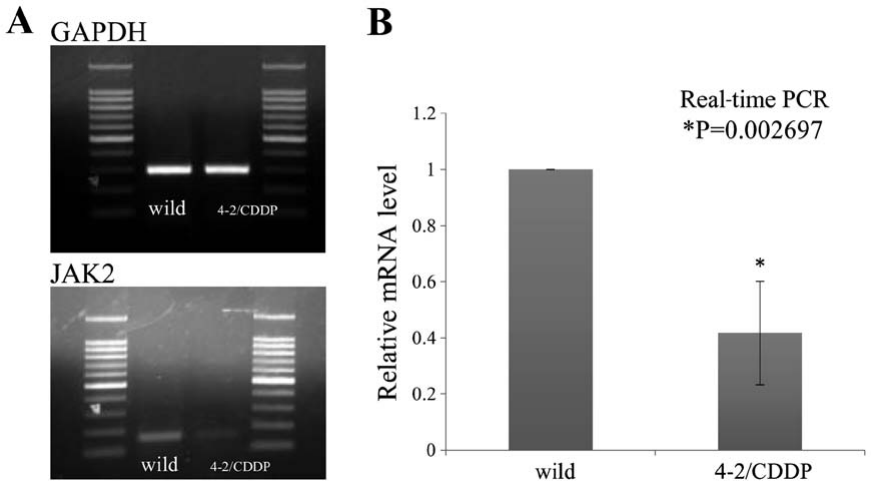
(A) Comparison of JAK2 mRNA levels in wild-type and TE4-2 cells using agarose gel electrophoresis. (B) Bar graph demonstrating a comparison of JAK2 mRNA levels in wild-type and TE4-2 cells. The analyzed mPCR level of JAK2 was significantly lower in the TE4-2 cells compared with the wild-type cells (P=0.002697).

The mRNA expression of JAK2 was measured using 3 different primers. The measured mPCR level of JAK2 was lower in the TE4-2 cells compared with the wild-type cells as shown in Fig. 6.

## Discussion

CDDP induces tumor death by means of platinum complexes binding to and causing cross-linking of DNA, ultimately triggering apoptosis (18). Since CDDP is one of the key drugs in treating not only esophageal cancer but also the majority of other cancers, a number of studies have focused on CDDP-resistance as this remains a major obstacle for successful cancer therapy. The mechanisms of resistance to CDDP are multifactorial; indeed, many genes or gene products have been reported to be involved in CDDP resistance. For instance, *ATG7* (5), *ZNRD1* (7), *MRP1/MRP2* (9,10), and various other genes have all been associated with cellular resistance to CDDP. While *MRP1* has been identified in several studies, the correlation between its expression and sensitivity is still fairly modest (12). Indeed, at the present time, no gene has yet been adopted for clinical therapy, which therefore requires further investigation.

In this study, we were able to identify 37 candidate genes responsible for the resistance of CDDP in two cell lines (TE4 and TE15) derived from ESCC cells. As for TE4, *IL-6* was over-expressed whilst *RPTOR, JAK2, LRP1, OR5J2, CCNTNAP2* and protein CASP were downregulated. Six genes were identified as overexpressed in TE15 cells, including *TRIM16L, TRAV26-2, KIAA0368, AQP7, OR1N2* and *LRRC38*, while 24 genes were downregulated, including *CAMKMT, PDE8B, DENND3, ZFPM2, UMODL1, CAMSAP2, TNFSF4, ACSTN2 RFX3, GNA14, INTS9, RNF19a, THSD7B, PTBP1, MVB12B, HEPHL1, NELL1, FHAD1, PCDH7, ROBO2, RFPL3, MICAL3, RAB3B* and *SYT10*.

In order to confirm the experimental system to be reproducible, we focused on *JAK2*, as it has been reported to have association with tumor genesis in previous studies (19–21). Downregulation of JAK2 was identified by the screening, and we were able to observe lower JAK2 level in the CDDP-resistant colonies. The protein associates with cytokine receptors and is essential for signal transduction by mediating tyrosine phosphorylation. Recent studies have implicated the de-regulation of JAK2 kinase activity in a number of myeloproliferative diseases (19) which is due to chromosomal translocations in hematopoietic tumors and mutations within the pseudokinase domain. In particular, a somatic mutation in the JAK2 protein, V617F, was identified in myeloproliferative neoplasms, including 95% of patients with polycythemia vera and ~50% of patients with essential thrombocythemia and primary myelofibrosis (20). Moreover, it has been reported that mutant JAK2 enhanced the resistance to CDDP-induced DNA damage and also suppressed apoptosis (21).

The overexpression of the *IL-6* precursor was also identified from our screen. *IL-6* has been reported to be associated with disease progression and poor prognosis in patients with esophageal cancer (22). Indeed, Suchi *et al* showed using ESCC cell lines KYSE170 and TE13 that the expression of IL-6 in esophageal carcinoma cells may act as a resistance factor against CDDP-based chemotherapy or chemo-radio-therapy (23). These results may therefore indicate *IL-6* as a strong candidate gene for CDDP resistance in ESCC cells. The fact that *IL-6* was also identified as a drug-resistant gene in our study may further confirm this finding. We are therefore continuing with further experiments.

The data obtained through this transposon-based method confirms that transposon mutagenesis provides a powerful, adaptable, and cost-effective genetic approach for identifying resistant genes in ESCC cells. The piggyBac element is especially useful because it can be precisely excised from an insertion site, thus restoring the site to its pre-transposon state. Indeed, precise excision is particularly useful when transient transgenesis is required (15). Moreover, the insertion mutagenesis approach generates various kinds of mutations including activation, repression, and truncation and is also not limited by cell type. Application of this method by either overexpressing or downregulating target genes may indeed lead to effective genetic therapy.

This study has some limitations. First, *in vitro* culture of drug-resistant cell lines may not always reflect that occurring *in vivo* and in some cases may even contradict this. It is therefore necessary to expand our study to *in vivo* experiments to confirm our data. There is a sufficient possibility that CDDP resistance may be caused by a combination of multiple gene mutations; however, we believe that we will be able to identify the ‘key genes' involved in this process. Second, although this method enables us to perform a comprehensive screen of drug-resistant genes, our screening shows a limited range of candidate genes because of the relatively low effectiveness in establishing transposon-tagged cells. One solution to this problem would be using a vector with a higher efficiency.

However, even with these potential limitations, the data in our study allowed us to identify consistent patterns of gene expression in cells from ESCC tumors. The method is inexpensive, relatively simple, and capable of identifying activating- and disrupting-mutations, leading to comprehensive screening. In addition, our data identified not only new possible resistance genes but also strengthens the evidence for previously identified candidates. Moreover, this method is applicable in searching for resistance candidates in other anti-cancer drugs, molecular target drugs, radiation-resistant genes, and even the combination of these therapies. We therefore believe that our study should impact chemotherapy treatment in patients with ESCC.

In conclusion, we were able to identify 37 candidate genes from two cell lines derived from ESCC cells resistant to CDDP. The method used is inexpensive, relatively simple, and capable of introducing activating and de-activating mutations, allowing for drug-resistant genes to be identified. Our future research will continue to involve identifying resistant genes and performing experiments to confirm the acquisition of drug resistance.

## Figures and Tables

**Table I:** Candidate genes for CDDP in ESCC cells.

Cell	Identities	Gene	Overexpressed/downregulated
4-2	98%	RPTOR	Overexpressed
	82%	JAK2	Downregulated
4-4	100%	LRP1	Downregulated
	100%	OR512	Downregulated
4-7	97%	CNTNAP2 precursor	Downregulated
	96%	Protein CASP isoform	Downregulated
	93%	IL-6 precursor	Overexpressed
15-5	83%	CAMKMT	Downregulated
	90%	TRIM16L	Overexpressed
	78%	PDE8B	Downregulated
	83%	DENND3	Downregulated
	92%	ZFPM2	Downregulated
	80%	UMODL1	Downregulated
	86%	TRAV26-2	Overexpressed
	84%	CAMSAP2	Downregulated
	80%	TNFSF4	Overexpressed
15-8	100%	ASTN2	Downregulated
	100%	KIAA0368	Overexpressed
	94%	RFX3 isoform	Downregulated
	100%	AQP7	Overexpressed
	100%	GNA14	Downregulated
	100%	OR1N2	Overexpressed
	100%	INTS9 isoform	Downregulated
	100%	RNF19a	Downregulated
15-14	86%	TSHSD7B	Downregulated
	94%	PTBP1	Downregulated
	90%	MVB12B	Downregulated
	100%	HEPHL1 precursor	Downregulated
	82%	NELL1 isoform	Downregulated
	96%	FHAD1	Downregulated
	87%	PCDH7	Downregulated
	92%	ROBO2 isoform/precursor	Downregulated
	92%	RFPL3	Downregulated
15-15	92%	MICAL4	Downregulated
15-19	89%	LRRC38	Overexpressed
	89%	RAB3B	Downregulated
	100%	SYT10	Downregulated

A total of 39 candidate genes from 8 resistant colonies were identified. CDDP, cisplatin; ESCC, esophageal squamous cell carcinoma.

## Abbreviations

CMV: cytomegalovirus
ESCC: esophageal squamous cell carcinomas
TE: Tohoku esophagus
CDDP: cisplatin
JAK2: Janus kinase 2
5-FU: 5-fluorouracil
RTOG: radiation therapy oncology group
QOL: quality of life
ZNRD1: zinc ribbon domain-containing-1
PB: PiggyBac
DMEM: Dulbeco's modified Eagle's medium
FBS: fetal bovine serum
